# New Mechanism Proposed for the Base-Catalyzed Urea–Formaldehyde Condensation Reactions: A Theoretical Study

**DOI:** 10.3390/polym9060203

**Published:** 2017-06-02

**Authors:** Taohong Li, Ming Cao, Jiankun Liang, Xiaoguang Xie, Guanben Du

**Affiliations:** 1 The Yunnan Province Key Lab of Wood Adhesives and Glued Products, Southwest Forestry University, Kunming 650224, China; caominghappy@swfu.edu.cn (M.C.); liangjiankun@swfu.edu.cn (J.L.); 2 Key Lab for Forest Resources Conservation and Utilisation in the Southwest Mountains of China, Southwest Forestry University, Ministry of Education, Kunming 650224, China; 3 School of Chemical Science and Technology, Yunnan University, Kunming 650091, China; xgxie@ynu.edu.cn

**Keywords:** urea–formaldehyde, base-catalyzed, condensation, mechanism

## Abstract

Base-catalyzed urea–formaldehyde condensation reactions were investigated by using a quantum chemistry method. It was found that monomethylolurea or *N*,*N*’-dimethylolurea can produce the methyleneurea intermediate (–HN–CO–N=CH_2_) with the catalysis of base. The E1cb (unimolecular elimination of conjugate base) mechanism was identified for the formation of such an intermediate. The potential energy barrier was theoretically predicted to be 59.6 kJ/mol for the E1cb step, which is about half of that of previously proposed S_N_2 (bimolecular nucleophilic substitution) mechanism. In the subsequentcondensation reactions, Michael addition reactions that lead to different condensed structures can occur between the methyleneurea intermediate and the anions produced from methylolureas under alkaline conditions. Based on the theoretical calculations on the kinetics and thermodynamics of the selected reactions, the competitive formations of methylene linkages, ether linkages and uron were discussed in combination with our previous experimental observations.

## 1. Introduction

It is generally believed that base-catalyzed urea–formaldehyde (UF) reactions primarily form methylolureas, while condensations that form polymers can hardly occur [1,2]. Therefore, UF resin synthesis must experience acidic stage. However, it has been shown in our previous study that the condensations that form methylene ether linkage (–CH_2_–O–CH_2_) occurred fast at pH = 8~9 [3]. Specifically, about 25% formaldehyde was converted to the condensed ether methylene carbon. In contrast, methylene linkages (–NR–CH_2_–NR) were not observed when the F/U molar ratio was controlled to be 2/1. Differently, when the molar ratio was lowered to be 1/1, methylene linkages became competitive with ether linkages. To rationalize these results, we performed quantum chemistry calculations and a bimolecular nucleophilic substitution (S_N_2) mechanism was proposed. Based on the calculated potential energy barriers, the competitive relationship of different condensation reactions was discussed. However, all the S_N_2 reactions have energy barriers above 100 kJ/mol. Especially, the linear ether linkage (–CH_2_–O–CH_2_) which was experimentally found to be dominant condensed structure encountered a barrier above 120 kJ/mol. This is not consistent with its fast formation at 80 and 90 °C. Therefore, there should be other energetically more favorable mechanism.

It is a fact that the UF condensation reactions under alkaline conditions have not received enough attention during the past decades because they are believed to be very minor. However, another fact is that many studies have shown the strategies to synthesize phenol-urea–formaldehyde (PUF) [4,5,6,7] and melamine-urea–formaldehyde resin (MUF) [8,9,10,11] under alkaline conditions. It is very likely that UF, MF, and PF share a common condensation mechanism, but selectivity of different intermediates determines whether co-condensations can occur. Therefore, there is no excuse for us to ignore the alkaline UF reactions.

We recently confirmed the formation of quinonemethide intermediate for based catalyzed phenol-formaldehyde (PF) condensation reactions by employing quantum chemistry calculations [12]. Enlightened by the identified E1cb (unimolecular elimination of conjugate base) mechanism, we speculate a similar mechanism may also be applied to UF condensations. This mechanism may have a lower energy barrier than S_N_2. To the best of our knowledge, such a mechanism has never been mentioned in any literature. To avoid misleading results caused by the wrong mechanism, in this work we performed theoretical calculations to re-examine the based-catalyzed UF condensation reactions. In combination with our previous experiments [3], the competitive relationship of the involved base-catalyzed condensation reactions was discussed.

## 2. Quantum Chemistry Calculations

All the structures of the stationary points on the reaction potential energy surfaces (PES)—including reactants, intermediates, transition states, and products—were fully optimized by using density functional theory [13,14] method B3LYP [15,16,17] with the standard basis set 6–31 ++ G** [18,19,20,21]. The harmonic vibrational frequencies were calculated to characterize the nature of the stationary points and to obtain the zero-point energies (ZPE) which were used to correct the relative energies. Each transition state was identified as they have unique imaginary frequencies. For all the calculations, the self-consistent reaction field (SCRF) method was employed with Polarizable Continuum Model (PCM) [22,23,24] by defining water as the solvent to simulate the implicit solvent effects (Water: *ε* = 78.3553). All the calculations were carried out with GAUSSIAN 03 program package (Frisch 2003) [25].

The relative energies in the potential energy profiles show in this paper were obtained by the following formulas

*ΔE*(*TS*) = [(*E*(*TS*) + *ZPVE*(*TS*)] − [*E*(*R*) + *ZPVE*(*R*)] (1)

*ΔE*(*P*) = [(*E*(*P*) + *ZPVE*(*P*)] − [*E*(*R*) + *ZPVE*(*R*)] (2)

here, *E*(*TS*) is the total electron energy of a TS, while *E*(*R*) represents the total electron energy of a local minimum which is on the reactant direction (left side) of a TS. Thus, Δ*E*(*TS*) represents the energy barrier of a TS relative to the left local minimum on the PES. Similarly, *E*(*P*) is the total energy of electrons of a local minimum which is on the product direction (right side) of a TS, and Δ*E*(*P*) corresponds to the relative energy of a right local minimum with respect to a left local minimum. Note that the left local minimum can be initial reactants or reactant-like intermediates, and the right local minimum can be reaction products or product-like intermediates.

## 3. Results and Discussion

**Figure 1:**
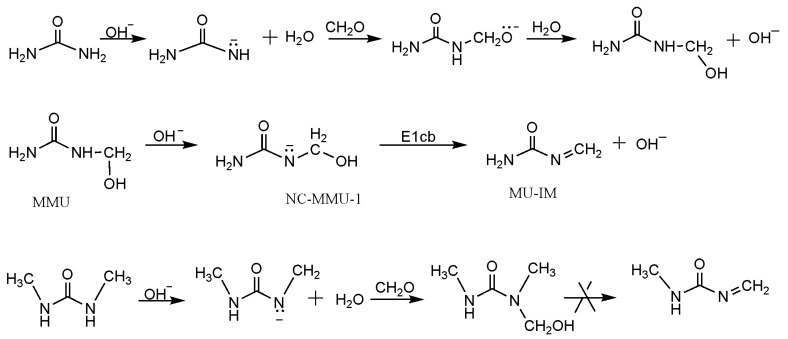
The mechanism proposed for the formation of methyleneurea intermediate.

According to the base-catalyzed quinonemethide intermediate formation from methylolphenol [12], we proposed the mechanism in Figure 1 where a methyleneurea intermediate (MU-IM) can be produced via E1cb elimination of OH^−^. An evidence that supports this mechanism is the condensation reactions of methylated dimethylureas were not observed under alkaline conditions [26,27] because methylol dimethylurea cannot produce methyleneurea intermediate according to this mechanism. To further confirm the validity of this mechanism, we performed theoretical calculations on the formation of MU-IM and the following condensations.

**Figure 2:**
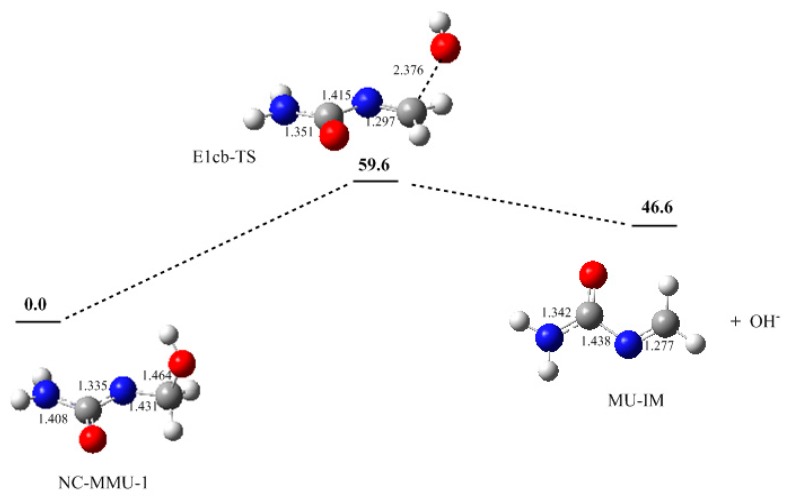
The potential energy profile (relative energy in kJ/mol) and the structures of the intermediates and transition state.

Here we modeled the formation of MU-IM from mono-methylolurea (MMU). In the first step, a proton on nitrogen atom is abstracted by OH^−^ due to its weak acidity, producing the nitrogen-charged intermediate MMU anion (NC-MMU-1). This is a fast reaction step. The following loss of conjugate base OH^−^ is the rate-determining step. As it can be seen in Figure 2, the transition state E1cb-TS has a potential energy barrier of 59.6 kJ/mol relative to NC-MMU-1. This barrier is much lower than that of the S_N_2 reaction (136.5 kJ/mol) [3], suggesting the E1cb mechanism is kinetically much more feasible. The formed MU-IM is less stable than NC-MMU-1 by 46.6 kJ/mol, indicating it is an unstable and reactive species. According to the theory of Michael addition [28,29], the reaction is characterized by nucleophilic addition of a carbanionor another nucleophile to an α,β-unsaturated carbonyl compound. As our calculations have confirmed that quinonemethide involved PF condensations belongs to this class. In fact, Michael addition belongs to the larger class of conjugate addition and therefore it should be safe to make an extension here since MU-IM has a similar structure with α,β-unsaturated carbonyl compounds as the N=CH_2_ conjugates with C=O.

**Figure 3:**
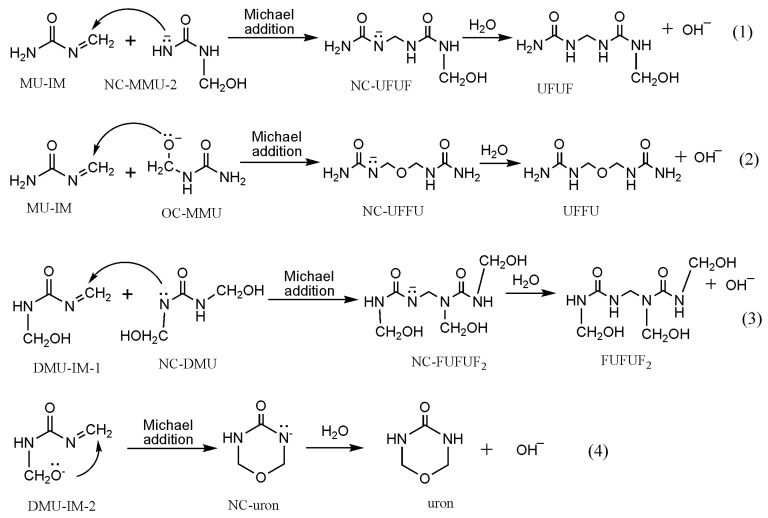
The typical condensation reactions based on the proposed mechanism.

**Figure 4:**
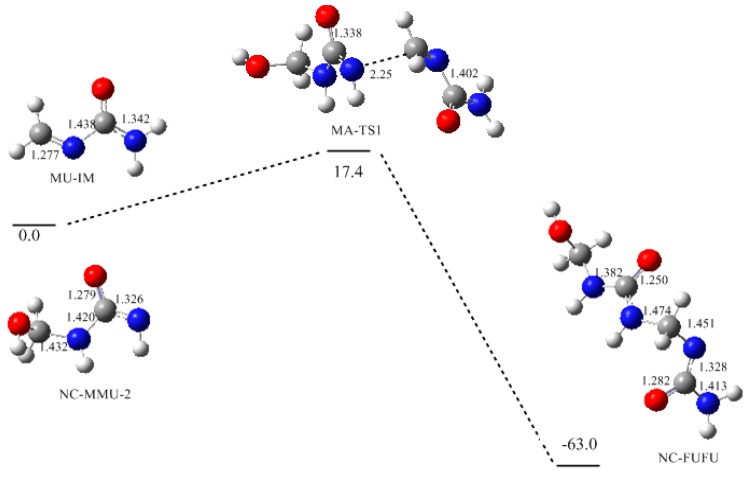
The potential energy profile (relative energy in kJ/mol) and the structures of the intermediates and transition state for reaction (1).

Based on the formation of MU-IM, we carried out further calculations on the typical condensation reactions shown in Figure 3 as (1)~(4). Reaction (1) demonstrates the Michael addition between the MU-IM intermediate and mono-methylolurea anion NC-MMU-2, which represents the formation of linear (un-branched) methylene linkage through the reaction of a free amino group and a methylol group in mono-substituted form. As shown in Figure 4, the Michael addition transition state MA-TS1 was successfully located indeed and the potential energy barrier represented by it was calculated to be 17.4 kJ/mol. This barrier is significantly lower than that of the E1cb formation of MU-IM, suggesting that the MU-IM formation is the rate-determining step while the followingcondensation reaction is faster. This is similar to the situation found for PF condensation [12]. With the formation of C–N bond, the Michael addition leads to the NC-FUFU intermediate which is more stable than the initial reactive intermediates by 63.0 kJ/mol. Fast protonation of this product-like intermediate finally produces the condensed dimer FUFU. 

**Figure 5:**
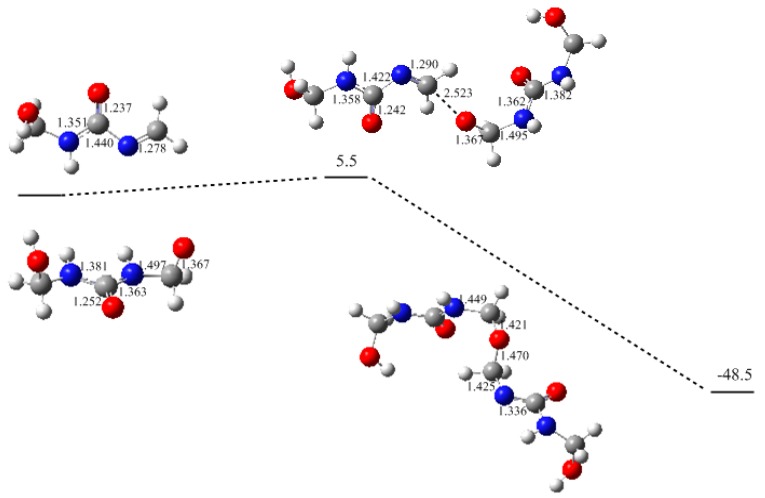
The potential energy profile (relative energy in kJ/mol) and the structures of the intermediates and transition state for reaction (2).

Reaction (2) corresponds to the formation of linear ether linkage. In this reaction, the oxygen-charged mono-methylolurea anion OC-MMU can be produced through the reaction of urea anion with formaldehyde, or through the reaction of methylolurea with OH^−^ as we have pointed out previously [3]. The calculated results in Figure 5 show that the Michael addition transition state MA-TS2 has a barrier of 8.1 kJ/mol relative to the initial intermediates. This barrier is only 50% of the barrier of reaction (1), indicating that the formation of linear ether linkage should be faster than formation of linear methylene linkage. However, they should be competitive processes since the barriers of the Michael addition step are lower than that of the rate-determining E1cb formation of MU-IM. This result can rationalize the observations of our previous experiments that the both the two types of linkages were formed when the F/U molar ratio was controlled to be 1/1. Specifically, the relative content of ether linkage (–NH–CH_2_–O–CH_2_–NH–) methylenic carbon calculated from quantitative ^13^C-NMR analysis was 24.5%, while the methylenic carbon of –NH–CH_2_–NH– accounts for 7.3% [3].

**Figure 6:**
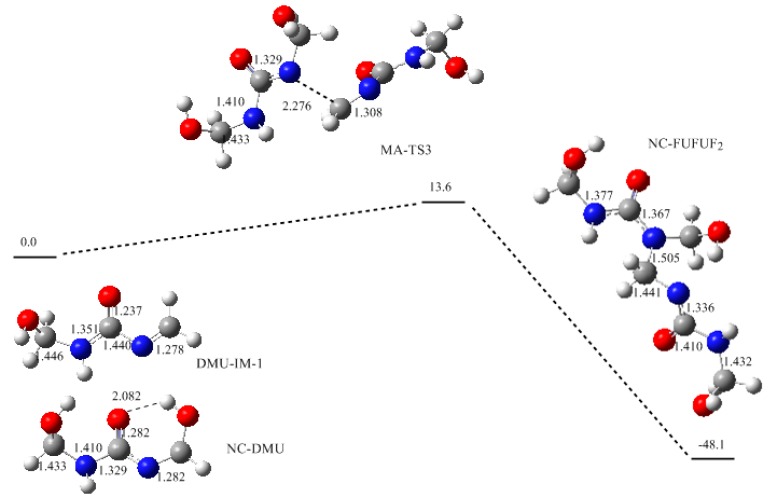
The potential energy profile (relative energy in kJ/mol) and the structures of the intermediates and transition state for reaction (3).

In the typical procedure of UF synthesis, the F/U molar ratio is generally controlled to be around 2/1. Under this condition, *N*,*N*’-dimethylolurea is the dominant monomer in condensations which produce branched methylene linkage (–N(CH_2_OH)–CH_2_–NH–) or linear ether linkage (–CH_2_–O–CH_2_–). However, with the 2/1 ratio, the methylene linkages were not observed within 90 min in our experiment [3]. They may have been formed but under detectable levels of ^13^C-NMR. Then it was expected that this reaction has a much higher energy barrier than the formation of ether linkage. The calculations based on the S_N_2 mechanism demonstrated that the potential energy barrier was indeed higher than that of ether linkage formation. However, the energy gap is not large enough to exclude formation of methylene linkage. Then we speculated that the steric hindrance may be another key factor that suppressed the formation of methylene linkages while the formation of ether linkages was not influenced by this effect. We believe this explanation can also be applied here although new mechanism was proposed. Because the substituted methylol group on nitrogen causes steric hindrance in the nucleophilic attack of nitrogen on another methylenic carbon, no matter in S_N_2 or in Michael addition. To confirm this, the reaction (3) in Figure 3 was also re-calculated according to the new mechanism and the results are shown in Figure 6. The barrier of this reaction was predicted to be 13.6 kJ/mol, slightly higher than that of reaction (2) by 5.5 kJ/mol, but lower than that of reaction (1) by 3.8 kJ/mol. Obviously, such a small difference cannot explain the absence of methylene linkages. Therefore, steric hindrance plays a more important role than potential energy barrier in determining the distribution of different condensed structures. In fact, the steric hindrance effect also successfully rationalized the competitive relationship between methylene and ether linkages under acidic conditions [30].

**Figure 7:**
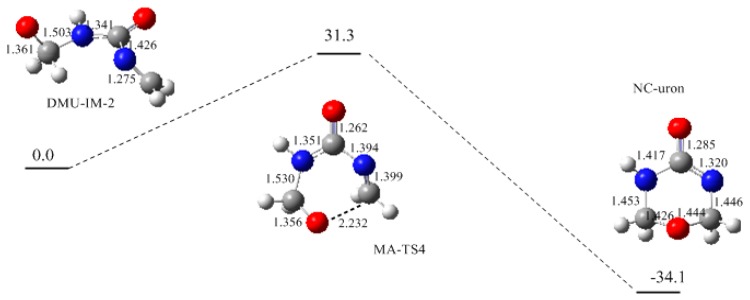
The potential energy profile (relative energy in kJ/mol) and the structures of the intermediates and transition state for reaction (4).

Intra-molecular Michael addition [28] of a di-methylolurea or tri-methylolurea can produce the cyclic ether structure, namely uron. The reaction (4) in Figure 3 shows the proposed mechanism. As it can be seen in Figure 7, this reaction has a barrier of 31.3 kJ/mol. This is the highest barrier among all the condensation reactions, but it is still lower than that of the rate-determining step. Moreover, this reaction is not affected by steric hindrance at all. Therefore, uron formation should be competitive with linear ether linkage. In our experiment (F/U = 2/1, 90 °C), the relative content of uron species accounted for 14.7%, next to linear ether linkage (25.3%). 

**Figure 8:**
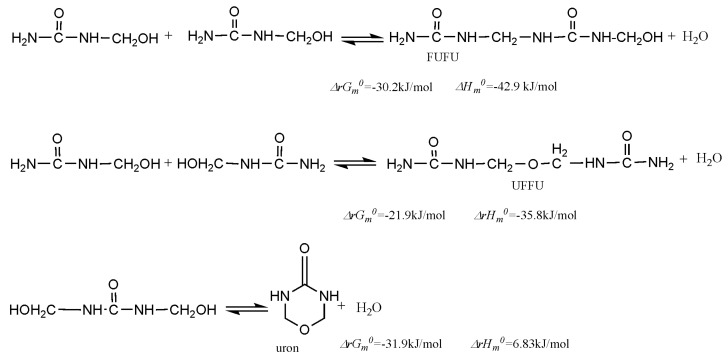
The calculated thermodynamic data for the condensation reactions.

All the above discussions are referred to the kinetics of the competitive reactions. However, the thermodynamic properties of them determine the distribution of their final products. Figure 8 shows the calculated thermodynamic data (298.15 K) for the selected reactions. It can be seen that the formations of methylene linkage and uron are thermodynamically more favorable than linear ether linkage, but at the initial stage of condensations, the system is far from chemical equilibrium and the reaction products are kinetically controlled. Therefore, the kinetically more favorable products appear to be dominant. As the reactions are undergoing and moving toward to equilibrium, the thermodynamically favorable products increase and become dominant. Therefore, the structures and components of the resin are dependent on when the reaction is stopped.

In the typical procedure of UF synthesis, the reaction time of initial weak alkaline stage (pH = 8~9) is generally less than one hour. Under this condition, the reactions mainly produce methylolureas and oligomers containing ether linkages and the typical resin structure cannot be formed. However, we should ask: what structures will be formed if the reactions are accelerated under strong alkaline condition? In fact, before this theoretical work, we did some experiments to find out what could be produced under strong alkaline conditions [31]. Under the conditions of F/U = 2/1, 90 °C, pH = 12~13, in the first hour of reaction, the contents of methylene carbon in different types of methylene linkages, ether linkages, and uron species were 1.1, 13.6, and 15.0%, respectively. Significant changes were observed when the reaction was stopped at 6.5 h. The values for the three structures became 43.2, 4.3, and 25.0%. Further, similar to the phenomenon observed in acid-catalyzed reaction, the conversion of ether linkages to methylene linkages was also observed. Obviously, as predicted by the theoretical calculations, the thermodynamically more stable structure became dominant even under alkaline conditions. These reactions were not as slow as we expected. This is another reason that urged us to re-consider the mechanism for the base-catalyzed UF reactions.

## 4. Conclusions

The base-catalyzed urea–formaldehyde condensation reactions were investigated by using quantum chemistry method at B3LYP/6–31 ++ G** level. The main conclusions have been drawn as follows: 

(1) Monomethylolurea or *N*,*N*’-dimethylolurea can produce the methyleneurea intermediate (–HN–CO–N=CH_2_) with the catalysis of base. The E1cb (unimolecular elimination of conjugate base) mechanism was identified for the formation of such intermediate. The potential energy barrier was theoretically predicted to be 59.6 kJ/mol, which is significantly lower than that of the previously proposed S_N_2 mechanism.

(2) The methyleneurea intermediate (–HN–CO–N=CH_2_) can be view as analogue of α,β-unsaturated carbonyl compounds. Therefore, Michael addition reactions occurring between the methyleneurea intermediate and the anions produced from methylolureas under alkaline conditions leads to different condensed structures.

(3) Based on the theoretical calculations on the kinetics and thermodynamics of the selected reactions, it was concluded that the formation of ether linkage is kinetically the most favorable process, while methylene linkage and uron are thermodynamically more favorable. The results of this work well rationalized previouslyobserved competitive formations of these condensed structures.
